# Neuropathology of Beta-propeller protein associated neurodegeneration (BPAN): a new tauopathy

**DOI:** 10.1186/s40478-015-0221-3

**Published:** 2015-06-30

**Authors:** R. Paudel, A. Li, S. Wiethoff, R. Bandopadhyay, K. Bhatia, R. de Silva, H. Houlden, J. L. Holton

**Affiliations:** Department of Molecular Neuroscience, UCL Institute of Neurology, London, UK; Reta Lila Weston Institute for Neurological Studies, UCL Institute of Neurology, London, UK; Sobell Department of Motor Neuroscience and Movement Disorders, Institute of Neurology, UCL, London, UK

**Keywords:** BPAN, WDR45, Tauopathy, Autophagy, Iron accumulation, NBIA

## Abstract

**Introduction:**

Beta-propeller protein associated neurodegeneration (BPAN) is associated with mutations in the WD repeat domain 45 (*WDR45*) gene on chromosome Xp11 resulting in reduced autophagic flux. This study describes the clinical and neuropathological features of a female 51 year old BPAN case. The clinical history includes learning disability and progressive gait abnormalities since childhood followed by progressive dystonic features in young adulthood. Brain imaging revealed generalised brain atrophy and bilateral mineralisation of the globus pallidus and substantia nigra.

**Results:**

The major pathological findings were observed in the substantia nigra with excess iron deposition, gliosis, axonal swellings and severe neuronal loss. Iron deposition was also observed in the globus pallidus. There was extensive hyperphosphorylated-tau deposition in the form of neurofibrillary tangles, pre-tangles and neuropil threads. Furthermore, histological studies and immunoblotting confirmed a mixed Alzheimer type 3-and 4-repeat tau pathology. Microtubule-associated protein 1A/1B-light chain 3 (LC3) immunoblotting of brain homogenates indicated autophagic activity and may support the role of WDR45 in autophagy.

**Conclusions:**

The widespread Alzheimer-type tau pathology in this disease indicates that this should be considered as a tauopathy and adds further support to the proposal that impaired autophagy may have a role in tauopathies.

**Electronic supplementary material:**

The online version of this article (doi:10.1186/s40478-015-0221-3) contains supplementary material, which is available to authorized users.

## Introduction

The dysregulation of brain iron metabolism leading to accumulation of iron in the basal ganglia and substantia nigra is a characteristic feature of the group of inherited neurological disorders classified under the term neurodegeneration with brain iron accumulation (NBIA). The main clinical features of NBIA include progressive dystonia, dysarthia, spasticity, parkinsonism and optic atrophy. The age of onset and rate of progression of the disease varies. Several genetic forms have been identified: pantothenate kinase-associated neurodegeneration (PKAN) with mutations in pantothenate kinase 2 (*PANK2*); PLA2G6-associated neurodegeneration (PLAN) due to mutations in phospholipase A2, group VI (*PLA2G6*), fatty-acid hydroxylase-associated neurodegeneration (FAHN) associated with mutations in fatty acid 2-hydroxylase (*FA2H*), mitochondrial membrane protein-associated neurodegeneration (MPAN) due to mutations in chromosome 19 open reading frame 12 (*C19orf12*), Kufor-Rakeb-Syndrome (KRS) due to ATPase type 13A2 (*ATP13A2*), aceruloplasminemia due to mutations in ceruloplasmin (*CP*), hereditary neuroferritinopathy due to mutations in ferritin-L subunit gene *FTL1*, Coasy protein-associated neurodegeneration associated with mutations in bifunctional coenzyme A synthase (*COASY)* and Woodhouse-Sakati-Syndrome (WSS) due to mutations in *DCAF17* [1, 2]. The mutations associated with different forms of NBIA involve a variety of metabolic pathways. *PKAN*, *PLA2G6*, *COASY* and *FA2H* have roles in fatty acid metabolism, *C19orf12* encodes a protein with a role in mitochondrial function and fatty acid metabolism. *CP* and *FTL1* have functions in iron homeostasis, *DCAF17* regulates transcription and *ATP13A2* is involved in apoptosis and magnesium metabolism [1, 2].

Recently *de novo* mutations in the gene encoding WD repeat containing protein 45 (*WDR45*) have been associated with an X-linked dominant form of NBIA causing static encephalopathy of childhood with neurodegeneration in adulthood (SENDA), now reclassified as BPAN (β-propeller protein-associated neurodegeneration), or NBIA5 [3–5]. Clinical manifestations include global developmental delay with intellectual disability in infancy or early childhood followed by further neurological and cognitive decline in adolescence with progressive dystonia, parkinsonism, ocular defects and sleep perturbation [3, 5]. During the course of clinical deterioration, iron accumulation involving the substantia nigra and globus pallidus is evident as observed in brain imaging [5]. *WDR45* encodes a beta-propeller scaffold protein which has a role in autophagy and autophagic flux has been found to be reduced in lymphoblasts from BPAN patients [4, 6].

The neuropathological description of a previously reported case with mutation in *WDR45* demonstrated the hallmark feature of NBIA, with iron accumulation in the substantia nigra and in the globus pallidus together with axonal spheroids, gliosis and severe neuronal loss. In the cerebellum significant depletion of Purkinje cells with axonal torpedoes was observed. Tau positive neurofibrillary tangles (NFTs) were described in several brain regions [5].

We report the detailed neuropathological description of a case with the previously reported loss-of-function mutation, p.Leu232Alafs*53 in *WDR45* [3, 5]. Tau pathology has been analysed using immunohistochemistry (IHC) and immunoblotting. We have also investigated autophagy in post-mortem brain tissue using immunoblotting for LC3.

## Materials and methods

### Patient and neuropathology

The brain was donated to the Queen Square Brain Bank for neurological disorders (QSBB) where all cases are collected for research according to ethically approved protocols and are stored under a licence from the Human Tissue Authority. The left half of the brain was fixed in 10 % neutral buffered formalin before being dissected in the coronal plane, examined and blocks selected for paraffin wax embedding and histology.

Paraffin-embedded sections (8 μm) were cut and stained using routine histological methods. Sections were stained using haematoxylin and eosin (H&E), Gallyas silver and Luxol fast blue/cresyl violet. IHC staining was performed using an avidin-biotin complex protocol using the antibodies and pre-treatments detailed in Additional file 1: Table S1. Antibody binding sites were visualized using the chromogen diaminobenzidine and sections were counterstained using Mayer’s haematoxylin.

Neuronal loss was assessed on H&E stained sections and scored semi-quantitatively (− = none; + = mild; ++ = moderate; +++ = severe). Tau pathology was assessed using AT8 antibody and the tau burden was scored semi-quantitatively for tau positive neuropil threads, pre-tangles and NFTs (− = none; + = infrequent; ++ = moderate; +++ = frequent).

### Immunoblotting

#### Tau

Sarkosyl-insoluble tangle PHF-tau was isolated from brain samples as previously described [7]. Insoluble PHF-tau was enriched from frontal cortex of the WDR45 case. As controls, a case each of Alzheimer’s disease (AD) and Pick’s disease (PiD) were selected from the archive of the QSBB [8, 9]. The homogenate was denatured in NuPage® LDS Sample buffer (Invitrogen, UK) at 70 °C for 10 min and loaded onto a 4–12 % Bis-Tris gel (Invitrogen, UK). The protein was then transferred onto nitrocellulose membrane (Amersham) and the membrane was incubated with antibody against tau (rabbit polyclonal, Dako) overnight at 4 °C followed by incubation for 1 h with an appropriate fluorescent secondary antibody and analysed using an Odyssey scanner (Licor).

#### Autophagosome associated light chain 3 (LC3)

Samples of frontal cortex from the WDR45 case and three age and sex matched controls selected from the archive of the QSBB were homogenised in 50 mM Tris–HCl pH7.5, 150 mM NaCl, 5 mM EDTA, 1 % tritonX-100, 2 % SDS containing complete protease inhibitor and phosphatase inhibitor (Roche). Tissue lysates were obtained after centrifugation of homogenised samples at 10000 g for 15 min. Protein concentration was determined using Bio-RAD protein assay kit. Protein lysates (40 μg) were analysed by SDS-PAGE on 4–12 % Bis-Tris gel (Nupage, Invitrogen) and transferred onto nitrocellulose membranes (Amersham). Membranes were blocked in 5 % BSA in phosphate buffered saline-tween for 1 h at room temperature, before being probed overnight at 4 °C with LC3 and beta-actin primary antibodies. The following steps of the protocol were as described above. The immunoblots were repeated twice with similar results.

## Results

### Clinical details

The female patient had learning difficulties as a child with progressive gait abnormalities. In school, other children would tease her for her “funny gait” as well as for being unable to keep up in class. By the age of 13, she moved to a special school for handicapped children. At age 19, she left school and continued to live in a specialised community for mentally and physically challenged people. Due to increasing behavioural difficulties at age 36 she moved to a social service hostel from where she attended a day centre several times a week.

Her movement disorder symptoms started deteriorating significantly at the age of 29 with slowing of gait and reduction of arm-swing whilst walking. Even though there were initial doubts in the diagnosis of Parkinson’s disease, she was started on Pergolide and later on Levodopa, both with equivocal and unpredictable effects with the subsequent development of dyskinesias and severe fluctuation of mobility. She deteriorated rapidly and developed dystonic features with an inward turning of the left foot, a bilateral akinetic-rigid syndrome with orofacial stereotypies during her thirties. Due to increasing behavioural problems, social withdrawal, reduced attention span and motivation a combination of cognitive and behavioural therapy was started in 1998. She was treated with Sertraline, and later with Amitriptyline, Velofaxine, Slisulpiride, and Carbamazepine, all with little effect. She had disturbed sleep with early morning awakening and behavioural problems with long off-phases. In the later stages of the disease she was wheelchair-bound and had urinary and faecal incontinence.

Other medical problems included slowly evolving difficulties with her eyesight with intermittent double-vision. She had a right corneal graft at the age of 32 – unfortunately no detailed further information on the underlying pathology of this was available. There was no family history except her mother who received a diagnosis of bipolar disorder and schizophrenia and died of a sudden brain haemorrhage when the proband was in her twenties.

Brain computerised tomography showed abnormal bilateral generalised mineralisation of the globus pallidus and substantia nigra, likely to represent iron deposition on subsequent MRI. Other investigations were normal except for WDR45 gene sequencing which showed a hemizygous mutation (c.694_703delCTGCGCCGAG, p.Leu232Alafs*53).

### Neuropathology

**Fig. 1:**
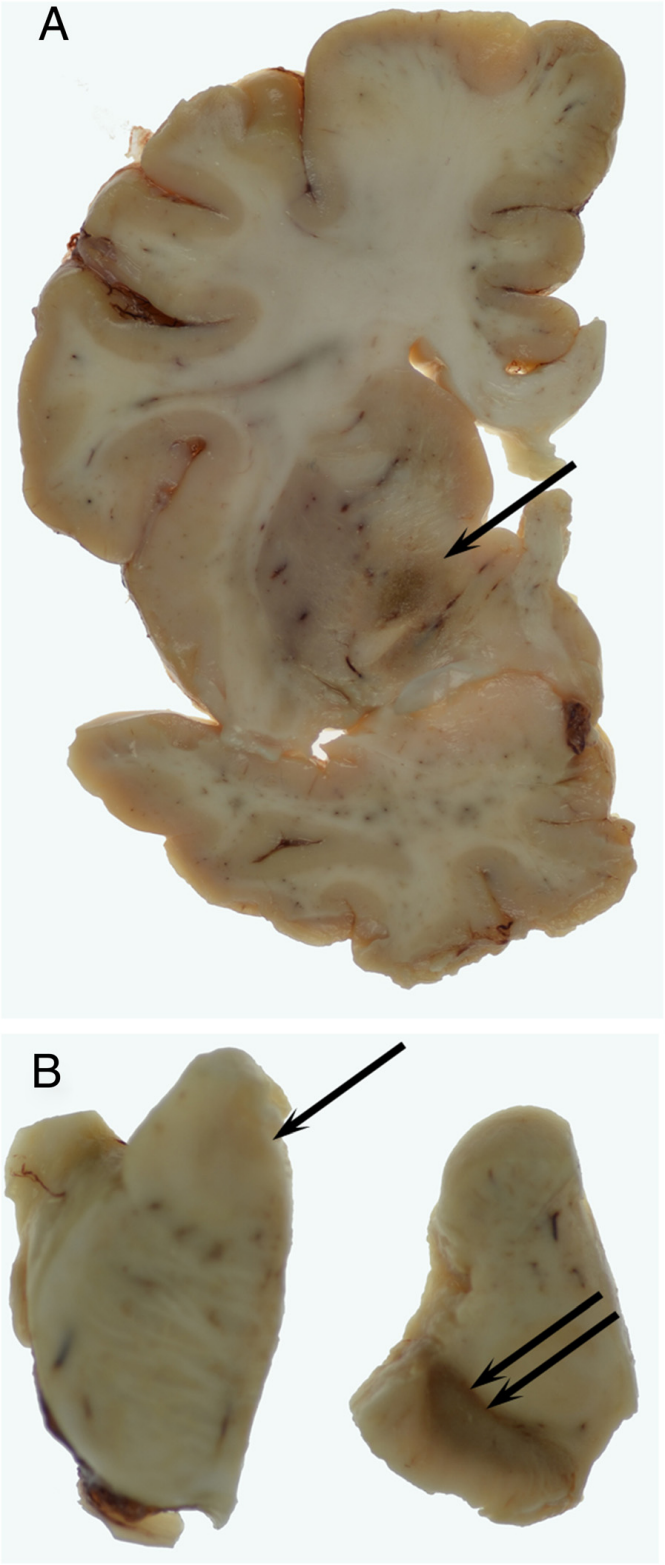
A coronal slice at the level of the anterior commissure shows atrophy and dark discolouration of the globus pallidus (**a**, arrow). There was loss of pigment in the locus coeruleus which was indiscernible (**b**, arrow). The substantia nigra was markedly abnormal with brown discolouration and a gelatinous texture (**b**, double arrow)

The fresh brain weighed 1,117 g. External examination of the left half brain showed frontal atrophy with preservation of parietal, temporal and occipital lobes. Coronal slices revealed mild dilatation of the frontal horn of the lateral ventricle and patchy thinning of the cortical ribbon in the frontal lobe. In the occipital cortex an area of cavitation with the appearance of an old infarct involved the primary visual cortex. The white matter in the anterior frontal and temporal lobes showed mild atrophy. The globus pallidus was reduced in size with blurring of the distinction between internal and external parts and showed dark discolouration anteriorly (Fig. 1a, arrow). The caudate and putamen appeared normal. The thalamus and hippocampus showed mild atrophy and there was moderate atrophy of the amygdala. There was marked abnormality of the substantia nigra which showed brown discolouration and had a gelatinous texture throughout (Fig. 1b, double arrow). The locus coeruleus was indiscernible (Fig. 1b, arrow). The medulla and cerebellum appeared normal.

**Fig. 2:**
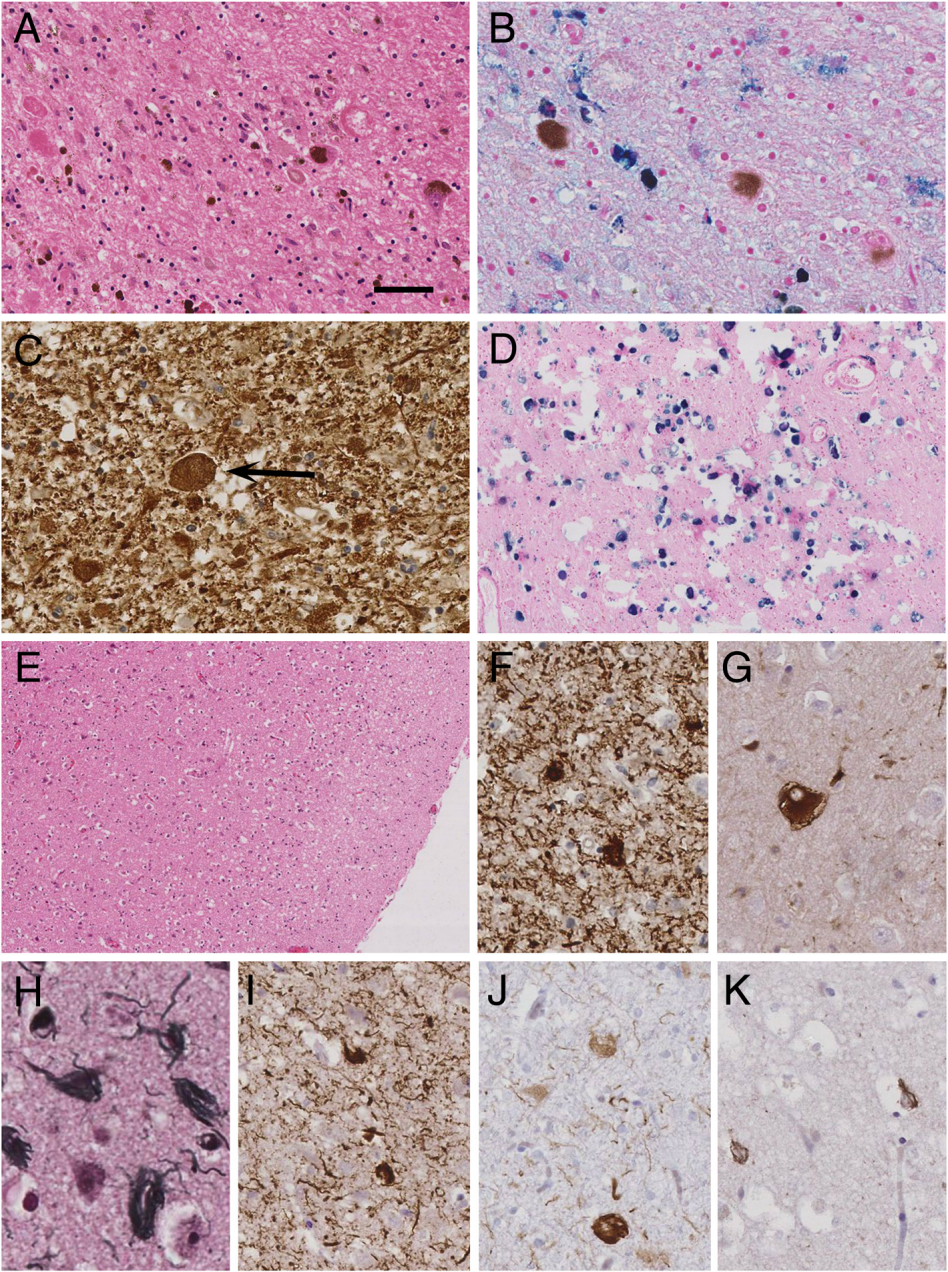
Histological features in *WDR45* mutation. In the substantia nigra there was severe loss of pigmented neurons with gliosis, axonal spheroids (**a**) and iron deposition (**b**). Axonal spheroids were highlighted using immunohistochemical staining for phosphorylated neurofilaments (**c**, arrow). There was also extensive iron deposition in the globus pallidus (**d**). In the frontal cortex there was mild neuronal loss with superficial spongiosis (**e**), phosphorylated tau accumulation was abundant in the form of threads and neurofibrillary tangles (**f**) and there were occasional balloon neurons. Fibrillar tau in neurofibrillary tangles was demonstrated by argyrophilia (**h**) and immunohistochemistry using AT100 confirmed the presence of paired helical filament tau (**i**). The 3-repeat (**j**) and 4-repeat (**k**) tau isoform specific antibodies illustrate the contribution of both groups of tau isoforms. **a** & **e** haematoxylin and eosin; **b** & **d** Perl’s stain; **c** SMI31; **f** AT8; **g** αB crystallin; **h** Gallyas silver impregnation; **i** AT100; **j** 3R tau; **k** 4R tau. Bar in **a** represents 200 μm in **d** & **e**, 80 μm in **a**, 40 μm in **b**, **c**, **f**, **g** & **i**–**k**

Histological examination (Fig. 2) showed mild superficial vacuolation in the anterior frontal, temporal and parietal cortices. A full thickness cortical infarct with cavitation, infiltration of macrophages and surrounding gliosis was confirmed involving the primary visual cortex. Neuronal loss was mild in neocortical regions (frontal, temporal, parietal, Table 1) and occasional balloon neurons were identified using a αB-crystallin antibody. Luxol fast blue staining confirmed good myelin preservation in the subcortical white matter. There was no cortical iron deposition identified using Perl’s stain.

**Table 1:** Summary of neuronal loss and semi-quantitative assessment of tau pathology in different brain regions

Brain regions	Neuronal loss	Tau pathology		
		Threads	Pre-tangles	Neurofibrillary tangles
Cortex				
Frontal	+	+++	-	++
Temporal	+	+++	-	++
Parietal	+	+++	-	++
Occipital	Infarct visual cortex	+	-	-
Cingulate	-	+++	-	+++
Sub-cortical white matter				
Frontal	N/A	+	N/A	N/A
Temporal	N/A	+	N/A	N/A
Parietal	N/A	+	N/A	N/A
Occipital	N/A	+	N/A	N/A
Cingulate	N/A	++	N/A	N/A
Amygdala	-	+++	-	+++
Hippocampus				
Dentate fascia	-	+	-	++
CA4	-	++	+	++
CA3	-	++	-	++
CA2	-	++	-	++
CA1	-	+++	+	+++
Subiculum	-	+++	+	+++
Entorhinal cortex	-	+++	+	+++
Transentorhinal cortex	-	+++	+	+++
Caudate	-	+++	+	++
Putamen	-	++	+	++
Globus pallidus	+	++	-	+
Meynert	+	+++	-	+++
Thalamus	-	++	-	++
Subthalamic nucleus	-	++	-	++
Substantia nigra	+++	++	-	+
Locus coeruleus	+	++	-	+
Pontine tegmentum	-	++	-	++
Pontine nuclei	-	+	-	+
Dorsal motor nucleus of vagus	-	+	-	+
Twelfth nerve nucleus	-	+	-	-
Inferior olive	-	+	-	-
Gracile nucleus	-	+	-	-
Cuneate nucleus	-	+	-	-
Cerebellar Purkinje cells	+	-	-	-
Cerebellar white matter	N/A	-	N/A	N/A
Dentate nucleus	-	+	-	+

*N/A* not applicable

Neuronal populations in the hippocampus, amygdala, striatum, thalamus and subthalamic nucleus were well preserved. Examination of the globus pallidus showed extensive mineralisation of vessels including arteries, veins and capillaries. Perl’s staining for iron was positive in areas of mineralisation and also in scattered cells with macrophage morphology. IHC staining for glial fibrillary acidic protein (GFAP) showed marked gliosis of the globus pallidus and there was also an increase in CD68-immunoreactive microglia. No axonal swellings were identified.

Examination of the midbrain showed very severe loss of pigmented neurons in the substantia nigra accompanied by gliosis, macrophage infiltration and marked deposition of Perl’s positive pigment. Numerous axonal swellings were present and were highlighted with IHC for amyloid precursor protein (APP), neurofilaments (neurofilament cocktail, SMI31) and ubiquitin. In the pons, the locus coeruleus showed mild neuronal loss with the pontine nuclei being well preserved. In the medulla the Xth and XIIth cranial nerve nuclei as well as the inferior olivary nucleus were well preserved. Axonal swellings were observed in the gracile and cuneate nuclei. The cerebellum showed mild Purkinje cell depletion with good preservation of the dentate nucleus and hemispheric myelin. There was no Aβ deposition in the hippocampus or neocortex. No α-synuclein pathology was found in brainstem or limbic regions and there was no TDP-43 pathology in limbic structures.

Tau pathology was extensive in the form of NFTs, pre-tangles and neuropil threads affecting cortical regions, white matter, deep grey nuclei, hippocampus, amygdala, brainstem structures and the cerebellum. Very rare coiled bodies were present in the sub-cortical white matter but there was no accumulation of tau in astrocytes. The type of tau pathology and semi-quantitative analysis of the tau burden in different brain regions is summarised in Table 1. Immunoreactivity for AT100, which recognises Alzheimer–type paired helical filament (PHF) tau, was also demonstrated. NFTs and neuropil threads were also identified using Gallyas silver staining further confirming the presence of fibrillar tau.

#### Tau isoform determination

**Fig. 3:**
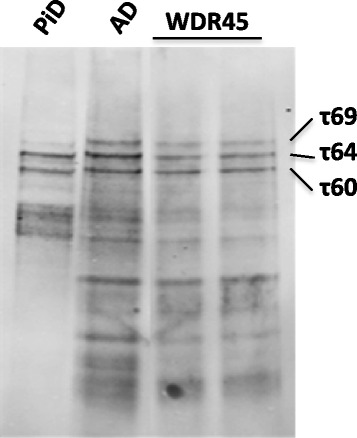
Western blot of non-dephosphorylated sarkosyl-insoluble brain fractions from Pick’s disease (PiD), Alzheimer’s disease (AD) and the *WDR45* mutation case which was run as duplicate samples. The *WDR45* case shows the classical triplet band as observed in AD cases due to mixed 3R- and 4R-tau pathology

Immunohistochemial staining using isoform specific antibodies demonstrated the expression of both 3- and 4-repeat tau isoforms. Immunoblotting of tau showed a pattern similar to that of AD with the classical triplet band of 3R- and 4R- tau isoforms compared to the 3R- tau isoforms predominantly observed in PiD (Fig. 3).

#### BPAN and autophagy

**Fig. 4:**
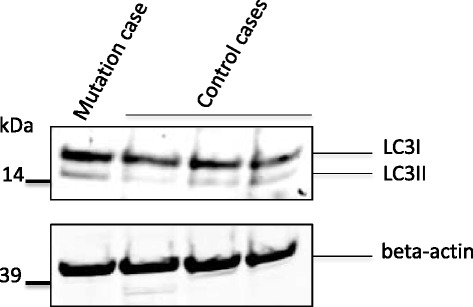
Immunoblotting of frontal cortical homogenates from the *WDR45* mutation case and three neurologically normal cases showing LC3I (~18 kDa) and LC3II (~16 kDa) levels (upper panel). Beta-actin loading control is shown in the bottom panel

In the mutation case and 3 controls LC3I and LC3II could be demonstrated by immunoblotting confirming autophagic activity (Fig. 4). As only a single case of *WDR45* mutation was available quantitation of immunoblots and statistical analysis was not performed and clear alteration of autophagic activity could not be demonstrated.

## Discussion

We report the neuropathological details of a 51 year old female with a clinical phenotype compatible with BPAN and positive for the hemizygous loss-of-function mutation, p.Leu232Alafs*53 in *WDR45*. This patient has previously been reported in genetic studies as case HH8 [3, 5].

The major pathological findings were severe neuronal loss with gliosis affecting the substantia nigra, iron deposition in the substantia nigra and globus pallidus, axonal swellings in the substantia nigra, gracile nucleus and cuneate nucleus together with very extensive phospho-tau deposition similar to that previously been reported [5]. We investigated the tau pathology, further characterising and documenting the distribution and abundance of neurofibrillary tangles, pre-tangles and neuropil threads. Furthermore, we demonstrated that the tau protein was in fibrillary conformation as structures were Gallyas silver positive and AT100 immunoreactive and both 3-and 4-repeat tau isoforms were represented similar to the tau deposits of AD. This finding was confirmed by immunoblotting of insoluble tau which showed a pattern similar to that of AD with the classical triplet band of 3R and 4R tau isoforms. This is a novel finding suggesting that BPAN may be considered as a tauopathy and further strengthening the link between tauopathies and other forms of NBIA such as PKAN, PLAN and MPAN in which a degree of neurofibrillary tangle pathology is a feature in some cases although extensive tau pathology is more often associated with PLAN [2, 10–13].

In neurodegenerative disorders, disruption of autophagy inhibits elimination of abnormal and toxic protein accumulation and can promote cellular stress and death. WDR45 belongs to a large family of WD40 repeat protein and is believed to be an important regulator of autophagy as documented in yeast [14] and *C. elegans* [15]. LC3-I is a soluble protein and conversion to LC3-II occurs during activation of autophagy where LC3-II is bound to the membrane of autophagosomes. An increase in LC3-II levels may indicate an increase in autophagy but also occurs where blockage in the final steps in autophagy impairs autophagic flux. Increased LC3-II and blockage of autophagic flux in lymphoblasts from patients with WDR45 mutation has been demonstrated implicating defective autophagy in the disease pathogenesis [4]. Although our results relate to only one patient and are therefore of a qualitative nature, they support the concept that dysfunctional autophagy is associated with *WDR45* mutation. As autophagy is known to contribute to the degradation of tau it seems likely that impaired autophagy in BPAN may underlie the severe tau pathology seen in this disease and supports the notion that autophagy may be important in the pathogenesis of other forms of tauopathy [16].

## Conclusion

We have described a case of BPAN and documented the neuropathological changes confirming a characteristic pattern of neuronal loss, most severe in the substantia nigra with iron deposition in both the globus pallidus and substantia nigra. Axonal swellings were present in the substantia nigra, gracile nucleus and cuneate nucleus. Impaired autophagic flux could not be demonstrated in a single case however, this could be the basis of future investigations. The striking widespread tau deposition was shown for the first time to be composed of fibrillar tau of Alzheimer-type and suggests that this disease may be considered as a tauopathy.

## Additional file

Additional file 1: Table S1. Primary and secondary antibodies used in IHC stainings and western blot.

## Abbreviations

BPAN: Beta-propeller protein associated neurodegeneration
WDR45: WD repeat domain 45
LC3: Microtubule-associated protein 1A/1B-light chain 3
PKAN: Pantothenate kinase-associated neurodegeneration
PANK2: Pantothenate kinase 2
PLAN: PLA2G6-associated neurodegeneration
PLA2G6: Phospholipase A2, group VI
FA2H: Fatty acid 2-hydroxylase
MPAN: Mitochondrial membrane protein-associated neurodegeneration
C19orf12: Chromosome 19 open reading frame 12
KRS: Kufor-Rakeb-Syndrome
ATP13A2: ATPase type 13A2
CP: Ceruloplasmin
WSS: Woodhouse-Sakati-Syndrome
*COASY*: bifunctional coenzyme A synthase
NFTs: Neurofibrillary tangles
SENDA: Static encephalopathy of childhood with neurodegeneration in adulthood
IHC: Immunohistochemistry
H&E: Haematoxylin and eosin
AD: Alzheimer’s disease
PiD: Pick’s disease
GFAP: Glial fibrillary acidic protein
APP: Amyloid precursor protein
