# Supplementary material for: Neuropathology of Beta-propeller protein associated neurodegeneration (BPAN): a new tauopathy
Source: Acta Neuropathol Commun. 2015 Jun 30;3:39. doi: 10.1186/s40478-015-0221-3 (PMC4486689; doi:10.1186/s40478-015-0221-3)
Supplement: Additional file 1: Table S1. — Primary and secondary antibodies used in IHC stainings and western blot. [file 40478_2015_221_MOESM1_ESM.docx]

| Supplementary Table 1: Primary and secondary antibodies used in IHC stainings and western blot | | | | | |  |
| --- | --- | --- | --- | --- | --- | --- |
| **Antibody** | **Clonality** | **Supplier** | **Application** | **Pre-treatment** | **Dilution** | **Incubation time** |
| Tau (AT8) | Monoclonal | Source Bioscience, UK (90206) | IHC | Citrate Buffer | 1/600 | 1 hour |
| Tau (AT100) | Monoclonal | Autogen bioclear | IHC | Citrate Buffer | 1/1000 | 1 hour |
| β-Amyloid (Aβ) | Monoclonal | DakoCytomation,Denmark (M0872) | IHC | Formic acid and Citrate Buffer | 1/100 | 1 hour |
| α-synuclein | Monoclonal | VectorLaboratories, Burlingame, CA (VP A106) | IHC | Formic acid and Citrate Buffer | 1/50 | 1 hour |
| TDP-43 | Polyclonal | Protein Tech, Chicago, IL (12892-1-AP) | IHC | Citrate Buffer | 1/2000 | 1 hour |
| α-internexin | Monoclonal | Abcam (967654) | IHC | Citrate Buffer | 1/75 | 1 hour |
| αβ crystalline | Monoclonal | Novocastra (ABCRYS-512) | IHC | Citrate Buffer | 1:300 | 1 hour |
| GFAP | Polyclonal | DakoCytomation,Denmark (M0872) | IHC | Proteinase K | 1/1000 | 1 hour |
| CD68 | Monoclonal | Dako | IHC | Citrate Buffer | 1/150 | 1 hour |
| SMI31 | Monoclonal | Sternberger | IHC | Citrate buffer | 1:5000 | 1hour |
| Neurofilament cocktail | Monoclonal | Cappel | IHC | Citrate buffer | 1:20 | 1hour |
| Amyloid precursor protein (APP) | Monoclonal | ABCAM | IHC | Citrate buffer | 1:200 | 1hour |
| Ubiquitin | Polyclonal | Dako | IHC | Citrate Buffer | 1/200 | 1 hour |
| P62 | Monoclonal | Bio Sciences | IHC | Citrate Buffer | 1/100 | 1 hour |
| Tau 3R | Monoclonal | Courtesy of Dr. Rohan De Silva | IHC | Citrate Buffer | 1:100 | 1 hour |
| Tau 4R | Monoclonal | Courtesy of Dr. Rohan De Silva | IHC | Citrate Buffer | 1:100 | 1 hour |
| Tau (rabbit  anti-human) | Polyclonal | DakoCytomation,Denmark (A0024) | WB | N/A | 1:20000 | Overnight |
| LC3 | Polyclonal | Novus Biologicals | WB | N/A | 1:2000 | Overnight |
| Β-actin | Monoclonal | Sigma | WB | N/A | 1:10000 | Overnight |
| Biotinylated rabbit anti-mouse IgG | Monoclonal | DakoCytomation, Denmark (E0354) | IHC | N/A | 1:200 | 1 hour |
| Biotinylated swine anti-rabbit IgG | Polyclonal | DakoCytomation, Denmark (E0353) | IHC | N/A | 1:200 | 1 hour |
| IRDye800CW Donkey anti-Rabbit IgG (H+L) | Polyclonal | Li-Cor(926-32213) | WB | N/A | 1:10000 | 1 hour |
| IRDye800CW Goat anti-Rabbit IgG (H+L) | Polyclonal | Li-Cor(926-32211) | WB | N/A | 1:15000 | 1 hour |
| IRDye680CW Donkey anti-Mouse IgG (H+L) | Monoclonal | Li-Cor(926-68072) | WB | N/A | 1:15000 | 1 hour |
